# Hybrid Multimodal Imaging Synthons for Chemoselective and Efficient Biomolecule Modification with Chelator and Near-Infrared Fluorescent Cyanine Dye

**DOI:** 10.3390/ph13090250

**Published:** 2020-09-16

**Authors:** Ralph Hübner, Valeska von Kiedrowski, Vanessa Benkert, Björn Wängler, Ralf Schirrmacher, Roland Krämer, Carmen Wängler

**Affiliations:** 1 Biomedical Chemistry, Department of Clinical Radiology and Nuclear Medicine, Medical Faculty Mannheim of Heidelberg University, Theodor-Kutzer-Ufer 1-3, 68167 Mannheim, Germany; 2 Molecular Imaging and Radiochemistry, Department of Clinical Radiology and Nuclear Medicine, Medical Faculty Mannheim of Heidelberg University, Theodor-Kutzer-Ufer 1-3, 68167 Mannheim, Germany; Valeska.vonKiedrowski@medma.uni-heidelberg.de (V.v.K.); Bjoern.Waengler@medma.uni-heidelberg.de (B.W.); 3 Institute of Inorganic Chemistry, Heidelberg University, Im Neuenheimer Feld 274, 69120 Heidelberg, Germany; Vanessa.Benkert@med.uni-heidelberg.de (V.B.); kraemer@aci.uni-heidelberg.de (R.K.); 4 Department of Oncology, Division of Oncological Imaging, University of Alberta, 11560 University Avenue, Edmonton, AB T6G 1Z2, Canada; schirrma@ualberta.ca

**Keywords:** chelator, click chemistry, cyanine dye, ^68^Ga^3+^, GRPR affinity, multimodal imaging, NIR fluorescent dye, peptide carrier, peptide homodimer

## Abstract

The development of hybrid multimodal imaging synthons (MIS), carrying in addition to a chelator for radiometal labeling also a near-infrared (NIR) fluorescent cyanine dye was the aim of this work. The MIS should be introducible into biomolecules of choice via an efficient and chemoselective click chemistry reaction. After chemical optimization, a successful synthetic strategy towards such hybrid MIS was developed, based on solid phase-based synthesis techniques and applying different near-infrared fluorescent cyanine dyes. The developed hybrid agents were shown to be easily introducible into a model homobivalent peptidic gastrin-releasing peptide receptor- (GRPR)-specific carrier without forming any side products and the MIS as well as their bioconjugates were radiolabeled with the positron-emitter ^68^Ga^3+^. The hybrid multimodal agents were characterized with regard to their log*_D_*s, GRPR target affinities and photophysical characteristics. It could be shown that the properties of the bioconjugates were not per se affected by the introduction of the MIS but that the cyanine dye used and specifically the number of comprised negative charges per dye molecule can have a considerable influence on target receptor binding. Thus, the molecular toolbox described here enables the synthesis of tailored hybrid multimodal imaging synthons for biomolecule modification, meeting the specific need and envisioned application of the combined imaging agent.

## 1. Introduction

Multimodal imaging for medical diagnosis is beneficial as it enables the confirmation of results obtained from one imaging modality by another method, providing complementary imaging results [1]. If the strengths of individual modalities are combined, it allows for synergistic imaging thereby enhancing the accuracy of diagnosis in a shorter time period [2].

Nowadays, combinations of imaging modalities are widespread in clinical diagnostic imaging such as positron emission tomography/computed tomography (PET/CT), which has been used since the early 2000s to provide both morphological and metabolic information. PET/MRI (magnetic resonance imaging) has been used for over 10 years, and also yields functional information conjoined with a better soft tissue contrast compared to CT and the overall reduced exposure to radiation translates into improved patient safety. Other diagnostic modalities used in multimodal imaging are photoacoustic ultrasound imaging [3], single photon emission computed tomography (SPECT) [4], or optical imaging (OI), preferably in the near-infrared (NIR) spectral window of 650–900 nm [5]. 

**Figure 1:**
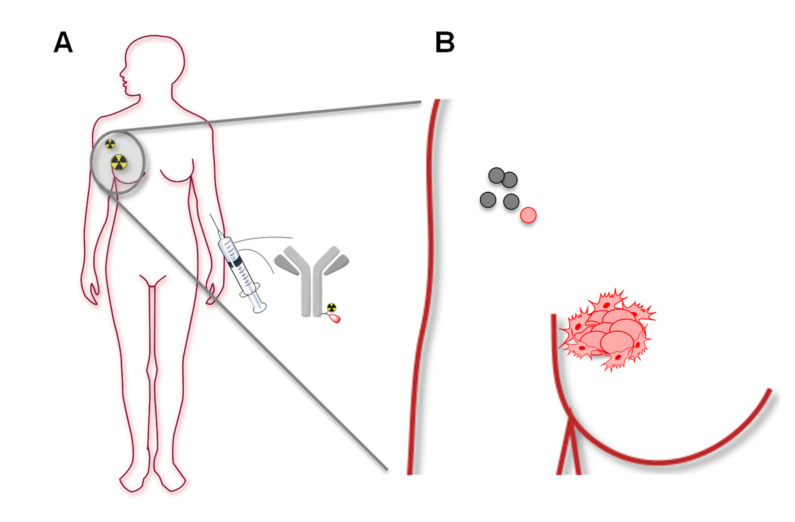
Schematic depiction of the operation principle of combined multimodal positron emission tomography/computed tomography (PET/CT) and near-infrared fluorescence optical imaging (NIR-OI) imaging. The first step is the injection of the multimodal, target-specific imaging agent and whole-body PET/CT imaging (**A**) followed by the intraoperative NIR-fluorescence optical image-guided surgery where the optically active compound clearly delineates e.g. tumor margins and identifies malignantly transformed lymph nodes (**B**).

Currently, one research focus is the combination of PET and near-infrared fluorescence optical imaging (NIR-OI) techniques because of the very high potential this particular combination offers. The excellent preoperative whole body PET imaging complemented by the possibility for intraoperative surgical guidance by NIR-OI is a unique feature of this union [6,7] (Figure 1).

Fluorescence-guided surgery is of high interest for the visualization of malignant tissue as the accurate detection of tumor borders and small metastases near the primary as well as malignantly transformed sentinel lymph nodes is important to help spare healthy tissue during the surgical removal. The high spatial resolution provided by NIR-OI guarantees a clear visual contrast between healthy and cancerous tissue [8,9,10]. Furthermore, the precise removal of cancer by accurate discrimination between tumor and non-cancerous tissue minimizes the risk of tumor recurrence and maximizes the speed of recovery for the patients [6]. Additionally, optical methods can further visualize tumor heterogeneity and cell surface biomarkers during histological characterization, being applied in optically-guided robotic needle biopsy [10] or in theranostic applications [11]. For intraoperative applications, a penetration depth of several millimeters of the emitted light is required. Common fluorescent dyes with absorption and emission properties in the visible spectral range do not meet this requirement as tissue and blood exhibit absorption and emission characteristics within the same wavelength range. However, molecules with fluorescent characteristics in the NIR-region, where autofluorescence of the tissue is not superimposing the emitted light, are better suited for this purpose.

Combined multimodal probes, comprising both the radiolabel and the fluorescent dye, exhibit a certain pharmacokinetic profile and guarantee the visualization of the same structures by both imaging modalities. In contrast, two separate agents, containing either radiolabel or dye in most cases exhibit a differing pharmacokinetic behavior, limiting the full comparability of the obtained imaging results. Thus, combined multimodal imaging agents are preferable to two different probes applied for PET or NIR-OI separately. However, the design of hybrid agents suitable for both imaging modalities requires the development of hybrid synthons that can be chemoselectively introduced into a target-specific carrier molecule. These agents have to comprise a positron-emitter for PET as well as an appropriate NIR fluorescent dye for NIR-OI.

In general, if a dual-labeling of biomolecules is performed, both labels are introduced separately, often resulting in impaired target binding of the carrier, especially in the case of relatively small biomolecules such as peptides or small proteins. However, clickable fluorescent hybrid synthons have been seldomly described [7,12,13,14,15] and no examples of NIR dye-carrying clickable multimodal imaging synthons (MIS) for PET/NIR-OI have been reported.

Thus, we aimed here at the development of a synthetic approach providing such hybrid NIR fluorescent dye-based multimodal imaging synthons that can be labeled with ^68^Ga or ^64^Cu and efficiently be introduced into a biomolecule of choice via a chemoselective click chemistry reaction.

## 2. Results and Discussion

### 2.1. Development of a Suitable Synthetic Pathway towards NIR Cyanine Dye- and Chelator-Comprising MIS Which Can Be Introduced into Biomolecules via Click Chemistry

**Figure 2:**
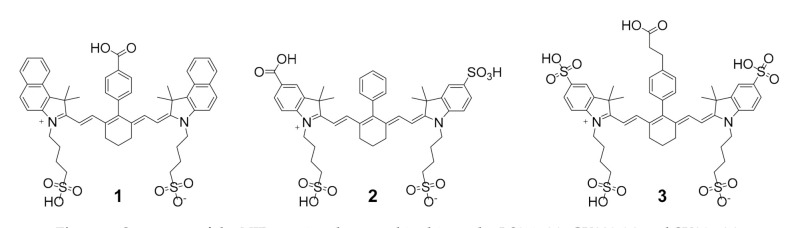
Structures of the NIR cyanine dyes used in this study: LS277 (**1**), CK002 (**2**) and SK015 (**3**).

When combining PET and NIR-OI, the PET-radionuclide in the molecular probe defines the synthetic strategy, because radiolabeling will always be the last step for molecules prepared for diagnostic PET imaging [7]. In this study, we chose to apply 1,4,7-triazacyclononane,1-glutaric acid-4,7-acetic acid (NODA-GA) as chelating agent as it allows the very efficient, mild and stable introduction of both positron emitters, ^68^Ga and ^64^Cu [16,17], exhibiting half-lives of 68 min and 12.7 h, therefore being appropriate for most PET imaging studies. As NIR fluorescent dyes, we chose to use three different cyanine dyes based on the parent compound indocyanine green (ICG), which has been in use for medical diagnosis over the last 60 years [5,18]. Specifically, we used the dyes LS277 (**1**) [19], CK002 (**2**) [20] and SK015 (**3**) [20] (Figure 2) as these three dyes span over a wide spectrum of properties, differing in the number of sulfonic-acid groups and thus charges, annulated rings as well as the conjugation point (center or edge of the molecule), influencing their steric demand when conjugated to smaller biomolecules such as peptides. The parent compound is well characterized in terms of medial use and exhibits a low toxicity and thus has only weak side-effects.

Apart from chelating agent and NIR fluorescent dye, the MIS were intended to comprise a functional group for mild, efficient, chemoselective and biocompatible introduction into a biomolecule of choice. For this purpose, we chose the well-established thiol-maleimide-based Michael addition reaction as it perfectly meets these criteria [21].

For the synthesis of these complex molecular systems, we developed a Fmoc-based solid phase-supported synthesis strategy to enable a high flexibility of the synthesis. Through this approach, the respective building blocks (e.g., chelator derivative or fluorescent dye) can be simply exchanged, allowing an easy adaption of the molecular design of the respective MIS to the specific application. Furthermore, solid-phase techniques allow to perform multiple reaction steps without purification after each conjugation reaction, making the whole synthesis process much faster and more efficient.

In order to synthesize the target MIS, we initially followed several different synthesis routes applying different solid supports, reaction conditions, coupling agents and positions for chelator and dye introduction, which, however, failed to give the intended products or resulted in suboptimal product yields. Details of these synthesis routes can be found in the supplementary information, including experimental details and supplementary Schemes S1–S3.

**Scheme 1:**
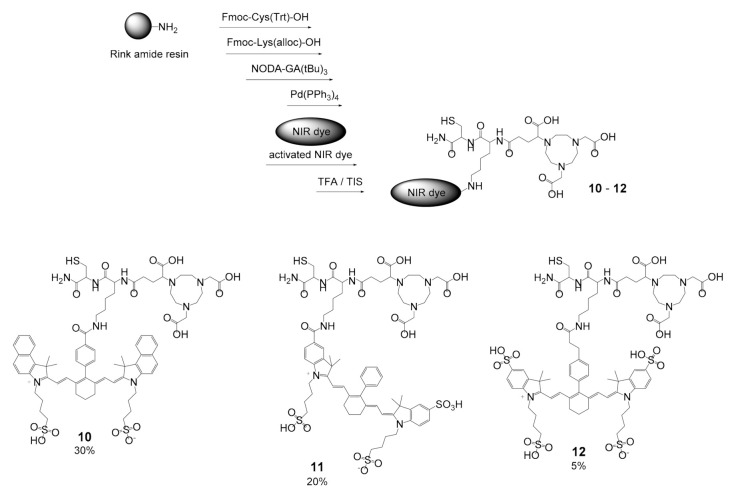
Schematic depiction of the final synthetic pathway towards the target multimodal imaging synthons (MIS) **10–12**.

Finally, we arrived to use Fmoc-Lys(alloc)-OH as orthogonally protected lysine derivative during the solid phase syntheses, enabling the chemoselective deprotection of the Lys-*N*_ε_ amino functionality using Pd(PPh_3_)_4_ without potentially compromising the integrity of the attachment of the whole molecules to the resin. As the solid support, we used a standard Rink amide resin and in a first step reacted it with cysteine to introduce the required thiol functionality into the molecule. This final synthetic route to the target MIS **10**–**12**, comprising the NIR cyanine dyes **1**–**3**, resulted in moderate to good overall synthesis yields of 5 to 30% and is depicted in Scheme 1.

A critical step during these syntheses of the MIS **10**–**12** was the conjugation of the cyanine dyes **1**–**3** to the Lys-*N*_ε_ amino functionality, where the complete dissolution of the dye molecules (at elevated temperature, ultrasound supported) in DMF (N,N-dimethylformamide) proved to be mandatory before activating their carboxyl function with HBTU (2-(1*H*-benzotriazol-1-yl)-1,1,3,3-tetramethyluronium hexafluorophosphate) and subsequent reaction on the resin. Furthermore, this reaction with the resin-bound material had to be carried out at an elevated temperature of 80 °C for three to four hours since performing the reaction at ambient temperature conditions did not result in product formation, even after prolonged reaction times of up to 24 h.

Taken together, the developed synthesis pathway towards the hybrid MIS was shown to be able to yield the target agents in high purity and reasonable yields. The synthesis is fully adaptable with respect to the introduction of the chosen chelating agent and NIR dye, therefore representing a general toolkit for MIS development.

### 2.2. Introduction of MIS 10–12 into a Model Biomolecule

In addition to the design and synthesis of the hybrid multimodal imaging synthons, their introduction into biomolecules is also of interest to determine the efficiency of conjugation and thus their general applicability for biomolecule modification. Furthermore, it is also important to determine the effect of such complex multimodal systems on the properties of the resulting bioconjugates. Thus, we aimed to demonstrate the introduction of the developed MIS **10**–**12** into a model bioactive compound to show their applicability for the synthesis of multimodal target-specific imaging agents.

As the hybrid multimodal imaging synthons are primarily intended to be used for the synthesis of tumor-specific imaging agents, a tumor targeting vector was chosen as a model biomolecule for MIS conjugation. For tumor targeting, mainly peptides and antibodies are used as they exhibit a high target affinity and specificity. Antibodies with their large size and complex molecular structure usually tolerate chemical modifications without considerably decreased target affinity. In contrast, the binding interaction of peptides with their specific biological targets can be significantly affected by even small chemical modifications. Furthermore, peptide modifications are usually defined in position and number of derivatization sites and the reactions can be easily followed by analytical methods. Thus, a peptide-based carrier seemed to be a reasonable choice for a model biomolecule to study MIS conjugation and determination of the influence of the MIS on the chemical, biological and photophysical characteristics of the resulting conjugates.

Specifically, we chose a homodimeric peptide shuttle targeting the gastrin-releasing peptide receptor (GRPR) for MIS conjugation. Radiolabeled GRPR-specific peptides have been the focus of radiochemical research for many years as this receptor type is highly overexpressed on a variety of tumors [4]. However, only one multimodal bombesin (BBN) analog has been reported for combined optical and photoacoustic imaging so far [22]. For GRPR-targeting, it was shown before that homodimers of GRPR-specific peptides enable an increased target affinity and tumor visualization sensitivity and thus exhibit superior properties compared to their monovalent counterparts [23,24]. We therefore chose this compound class for the determination of the conjugation efficiency with our newly developed MIS and determined the influence of the respective MIS on the chemical, biological and photophysical properties of the resulting bioconjugates.

**Figure 3:**
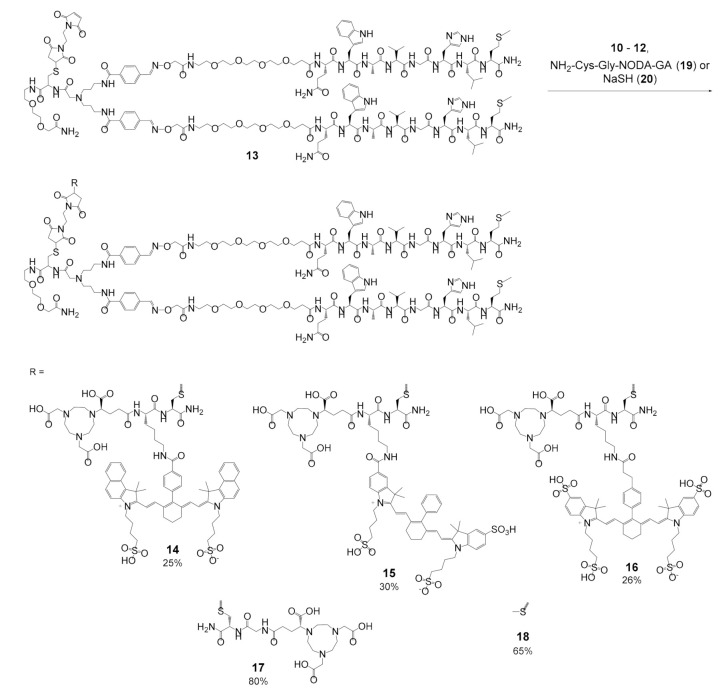
Structures of the chelator- and NIR-fluorescent dye-modified bioconjugates **14**–**16**, the chelator-only analog **17** and **18**, lacking both chelator and NIR dye.

For this purpose, we synthesized a maleimide-modified PESIN (PEG_3_-BBN_7–14_, where BBN_7–14_ is a truncated analog of the endogenous GRPR-binder bombesin (BBN)) homodimer (**13**) [13] as model target-specific agent for the following MIS conjugation reactions. The maleimide of **13** could be reacted with the thiols of MIS **10**–**12** efficiently and chemoselectively under mild reaction conditions (ambient temperature, neutral pH, within minutes) via Michael addition to the final NIR dye- and chelator-modified bioconjugates **14**–**16** (Figure 3). The products were isolated in good yields of 25–30% and no side product formation was observed.

In order to be able to study the influence of the chelator- and dye-comprising MIS and also of the single components of the hybrid agents on the mentioned properties of the resulting bioconjugates, we further synthesized two reference compounds: one comprised only the NODA-GA chelator but no additional cyanine dye (**17**) and the other comprised neither chelator nor dye (**18**) but only a small thio-methyl group to inactivate the maleimide of **13** and prevent its further reaction (Figure 3). Through this approach, the influence of each structure element of the MIS (chelator and dye) on the properties of their bioconjugates can be assessed separately. To obtain **18** and **17**, **13** was reacted with sodium methanethiolate (**20**) or NH_2_-Cys-Gly-NODA-GA (**19**), respectively. **19** was obtained analogously to the MIS **10**–**12** by solid-supported synthesis.

Thus, we showed that the synthesized MIS can be chemoselectively introduced into maleimide-comprising biomolecules with high efficiency, yielding the modified multimodal imaging agents in acceptable yields.

### 2.3. Determination of the ^68^Ga-Radiolabeling Efficiency of the MIS **10**–**12** and **19** and their Bioconjugates **14**–**17**: Influence of the MIS on the Chemical, Biological and Photophysical Properties of the Resulting Peptide-Based Bioconjugates

At first, we intended to determine the radiolabeling characteristics of the MIS **10**–**12** and the chelator-only analog **19**, as well as their respective bioconjugates **14**–**17** with the positron-emitter ^68^Ga^3+^. In particular, we were interested to see if the agents can be radiolabeled under standard conditions without the formation of side products or suffer from radiolysis-induced or thiol-associated instability.

**Figure 4:**
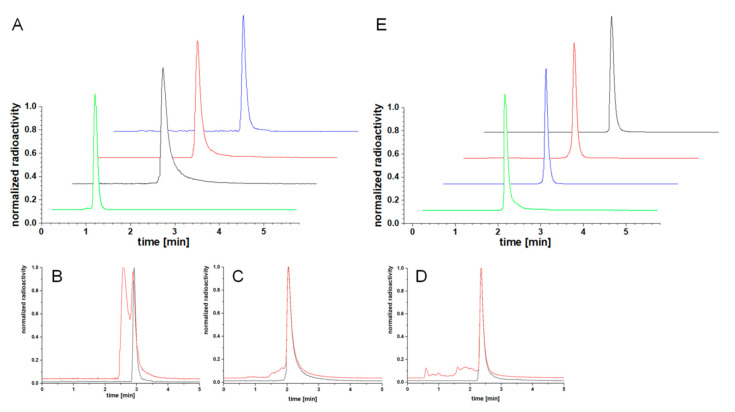
Radio-HPLC chromatograms of [^68^Ga]Ga-**10**–[^68^Ga]Ga-**12** and [^68^Ga]Ga-**19** ([^68^Ga]Ga-**10**: blue; [^68^Ga]Ga-**11**: black; [^68^Ga]Ga-**12**: red; [^68^Ga]Ga-**19**: green) (**A**). Radio-HPLC chromatograms of [^68^Ga]Ga-**10**–[^68^Ga]Ga-**12** from left to right; black traces: radiolabeling with tris(2-carboxyethyl)phosphine (TCEP), red traces: radiolabeling without TCEP (**B**–**D**). Radio-HPLC chromatograms of [^68^Ga]Ga-**14**–[^68^Ga]Ga-**17** ([^68^Ga]Ga-**14**: black; [^68^Ga]Ga-**15**: blue; [^68^Ga]Ga-**16**: red; [^68^Ga]Ga-**17**: green) (**E**). Analytical HPLC chromatograms were obtained using a gradient of 0% H_2_O–100% MeCN (+0.1% trifluoroacetic acid (TFA)) in 5 min.

When ^68^Ga-radiolabeling of **10**–**12** and **19** was performed under standard reaction conditions (aqueous solution at pH 3.5–4.2 without additives), heterogeneous products were obtained for [^68^Ga]Ga-**10**–[^68^Ga]Ga-**12** (Figure 4B–D). Furthermore, the extent of side product formation got more pronounced the longer the reaction mixtures were allowed to react. Adding an excess of the reducing agent tris(2-carboxyethyl)phosphine (TCEP), however, resulted in the disappearance of the side product species and the target ^68^Ga-MIS could be obtained in high radiochemical yields (RCY) and purities (RCP) of >95% under these conditions (Figure 4A).

These side product formations during the ^68^Ga-labeling of the thiol-comprising agents **10**–**12** can most probably be attributed to the dimerization of the agents, which could be reversed by TCEP. The dimerization under ^68^Ga-radiolabeling conditions was interestingly not observed for **19**, lacking the NIR dye, and homogeneous [^68^Ga]Ga-**19** was obtained with the same high RCYs and RCPs as [^68^Ga]Ga-**10**–[^68^Ga]Ga-**12** without the need to add TCEP to the reaction mixtures.

In addition to radiolabeling the MIS **10**–**12** and **19** with ^68^Ga^3+^, their bioconjugates **14**–**17** were also ^68^Ga-labeled. As these agents do not exhibit free thiol functionalities, the radiolabeling could be performed under standard radiolabeling conditions, not requiring TCEP to obtain uniform products. [^68^Ga]Ga-**14**–[^68^Ga]Ga-**17** could be obtained in high RCYs and RCPs of ≥95% (Figure 4E, Table 1, *n* = 3) and non-optimized molar activities of up to 156–198 GBq/µmol (164 GBq/µmol for **10**, 184 GBq/µmol for **11**, 156 GBq/µmol for **12**, 190 GBq/µmol for **19**, 198 GBq/µmol for **14**, 165 GBq/µmol for **15**, 165 GBq/µmol for **16** and 180 GBq/µmol for **17**), depending on the amount of starting activity. Furthermore, no degradation or rearrangement of the products was detected, neither during radiolabeling nor afterwards.

However, it has to be mentioned that an excess of ascorbic acid (2 mg per radiolabeling reaction) was mandatory to suppress radiolysis-induced fragmentation of the radiolabeled products, which can be attributed to the presence of the large, electron-rich dye molecules that are susceptible to oxidation processes.

The radiolabeled MIS and their corresponding bioconjugates were evaluated regarding their lipophilicity. Within the group of MIS, [^68^Ga]Ga-**10** exhibits a relatively high lipophilicity with a log*_D_* of −1.29 ± 0.11. In contrast, [^68^Ga]Ga-**11**, [^68^Ga]Ga-**12** and [^68^Ga]Ga-**19** demonstrated high hydrophilicities with log*_D_*s of −3.05 ± 0.08, −3.47 ± 0.17 and −3.35 ± 0.15, respectively. The relatively low water solubility of [^68^Ga]Ga-**10** can be attributed to the annulated aromatic rings of **1** (log*_D_* = −1.03 ± 0.11) and the lower number of only two sulfonic acid groups instead of three (**2**, log*_D_* = −4.00 ± 0.05) or four (**3**, log*_D_* = −3.95 ± 0.15).

These results indicate that the solubility properties of the used cyanine dyes dominate the log*_D_*s of the corresponding MIS. The same trend was observed for the multimodal imaging bioconjugates [^68^Ga]Ga-**14**–[^68^Ga]Ga-**17**, exhibiting log*_D_*s of −1.33 ± 0.04, −2.48 ± 0.14, −2.55 ± 0.13 and −2.38 ± 0.03, respectively (Table 1). The methylthioether equipped peptide homodimer **18**, lacking both chelator and cyanine dye (log*_D_* = −0.99 ± 0.03), exhibits a moderate solubility in the aqueous phase and indicates that the peptide shuttle also influences the water solubility of the bioconjugates.

However, the results show that the respective ICG dye molecule used has a more pronounced effect on the log*_D_* of the resulting bioconjugates than the bioactive carrier.

**Figure 5:**
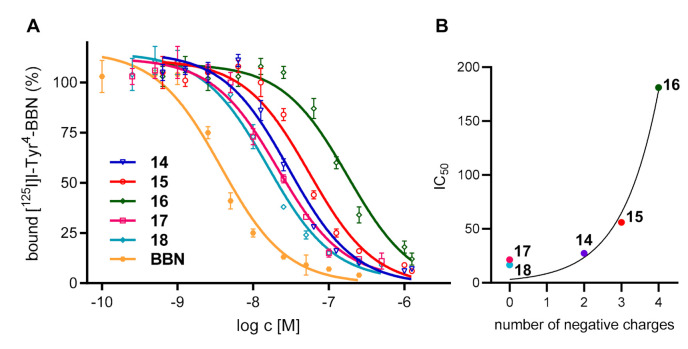
Results of the competitive displacement assays of the hybrid multimodal imaging synthon-bioconjugates **14**–**16**, the reference compounds **17** and **18** as well as bombesin (BBN) on stably GRPR-transfected human embryonic kidney 293 (HEK) cells using [^125^I]I-Tyr^4^-bombesin as competitor. The 50% inhibitory concentration (IC_50_) values for BBN (being used as internal standard with known high GRPR affinity) and **14**–**18** were determined to be 2.81 ± 0.56 nM, 27.39 ± 2.01 nM, 56.07 ± 1.47 nM, 181.23 ± 2.45 nM, 21.48 ± 1.2 nM and 16.64 ± 1.06 nM, respectively (**A**). Correlation between IC_50_ values and number of negative charges of **14**–**18** (**B**).

Another factor that could be influenced by the introduction of hybrid multimodal imaging synthons into biomolecule carriers is the target affinity of the receptor-affine agent. In order to determine the potential effect of the introduced MIS on the affinity of the peptide homodimers, GRPR-affinities were determined for the bioconjugates **14**–**18**. For this purpose, stably GRPR-transfected human embryonic kidney 293 (HEK-GRPR) cells were used and the target affinities were determined by competitive displacement studies using commercially available [^125^I]I-Tyr^4^-bombesin as competitor and endogenous bombesin as the internal standard with known high GRPR affinity. The 50% inhibitory concentration (IC_50_) values, being a measure for receptor affinity, were determined to be 2.81 ± 0.56 nM for BBN, 27.39 ± 2.01 nM for **14**, 56.07 ± 1.47 nM for **15**, 181.23 ± 2.24 nM for **16**, 21.48 ± 1.20 nM for **17** and 16.64 ± 1.06 nM for **18** (Figure 5A), being in accordance with previous affinity data obtained for PESIN homodimers [23,24].

From the IC_50_ values it can be deduced that the introduction of the NODA-GA chelator in the monomodal derivative **17** (21.48 ± 1.20 nM) only slightly altered the GRPR binding affinity compared to the PESIN homodimer lead **18** (16.64 ± 1.06 nM), comprising neither chelator nor cyanine dye. Likewise, the introduction of the additional NIR dye **1** decreased the target GRPR affinity of **14** only negligibly, pointing to a per se minor influence on binding affinity of even complex and bulky substituents in the chosen position of the peptide dimers.

A completely different observation was made when the ICG dyes **2** and **3** were conjugated instead of **1** (resulting in derivatives **15** and **16**). In these cases, a considerable loss of affinity could be observed ranging from **14** (27.39 ± 2.01 nM) over **15** (56.07 ± 1.47 nM) to **16** (181.23 ± 2.45 nM), respectively. The structural differences within the used cyanine dyes are not sufficient to explain the observed decreases in affinity as they exhibit a similar spatial demand. The major difference between the cyanine dyes used is the number of sulfonic acid groups and thus the higher negative charge of the conjugates containing **2** and **3** compared to **1** (Figure 5B).

A possible explanation for these results might be that the highly charged dyes interact with the peptide strands of the PESIN homodimer, resulting in an alteration of the secondary structure of the peptide units, going along with a loss of receptor interaction.

Finally, the photophysical properties were determined for dyes, MIS and their conjugates. It could be shown that the photophysical properties remained largely unaltered, including absorption coefficients and emission characteristics (Table 1). Neither loss nor significant shift of fluorescence properties were observed for the MIS **10**–**12** or their bioconjugates **14**–**16** compared to the dyes **1**–**3**. The only change in photophysical properties we qualitatively observed was an increase in the quantum yields of the dye conjugates compared to their unconjugated leads **1**–**3**, an effect that was described before and can be attributed to the conjugation of sterically demanding groups (such as the PESIN homodimers used here), resulting in a decrease in aggregation of the dye molecules and thus a reduced fluorescence quenching [25,26,27].

These results show that the introduction of the developed, NIR dye-comprising multimodal imaging synthons into maleimide-comprising biomolecules is easily via chemoselective ligation with high efficiency, without producing any side products. The resulting bioconjugates could be efficiently radiolabeled with ^68^Ga without producing side products, giving the products in high RCYs, RCPs and molar activities. Only the thiol-modified MIS have to be stabilized against dithiol-formation under radiolabeling conditions in case a two-step labeling approach was preferred (e.g., to increase radiolabeling efficiencies for large protein carriers).

The chemical, biological and photophysical properties of the resulting bioconjugates were generally preserved but depended on which cyanine dye was used for the synthesis of the MIS, whereas the introduced chelator had almost no effect.

## 3. Materials and Methods

### 3.1. General

All commercially available chemicals and solvents were at least of analytical grade and used, if not otherwise stated, without further purification. Fmoc-protected amino acids and resins were purchased from NovaBiochem. Fmoc-NH-PEG_3_-COOH, Fmoc-NH-PEG_1_-COOH, Fmoc-*d*-Cys(S-S-*t*Bu)-OH, (Boc)_2_AOAc-OH × H_2_O and Fmoc-NH-Propyl)_2_Gly-OH × KHSO_4_ were obtained from Iris Biotech, *N*-succinimidyl-4-formylbenzoate (SFB) and methyl mercaptan sodium salt from ABCR and tris(2-carboxyethyl)phosphine hydrochloride (TCEP) from Alfa Aesar. HBTU and trifluoroacetic acid (TFA) were purchased from Carl Roth; (N,N-diisopropylethylamine (DIPEA), triisopropylsilane (TIS), ascorbic acid and IR-820 from Sigma-Aldrich; 4-carboxyphenylboronic acid, 1,2-bis(maleimido)ethane (BME) and tetrakis(triphenylphosphine)palladium(0) (Pd(PPh_3_)_4_) from TCI and 4-(4,7-bis(2-(tert-butoxy)-2-oxoethyl)-1,4,7-triazacyclononan-1-yl)-5-(tert-butoxy)-5-oxopentan-oic acid ((*R*)-NODA-GA(*t*Bu)_3_) from CheMatech.

Dye derivatives **1** [19], **2** [20], **3** [20], Py1 [28] and 5(6)-carboxyfluorescein-pfp ester [29] were prepared according to literature methods. PESIN homodimer **13** was synthesized according to published procedures [13]. Syntheses on solid support were carried out applying standard Fmoc solid-phase peptide synthesis protocols [23,30], using standard commercially available resins, HBTU as coupling agent and *N*_α_-Fmoc-amino acids. The coupling reactions were carried out in DMF for 30 min using 4 eq. of amino acid, 3.9 eq. of HBTU as coupling reagent and 4 eq. of DIPEA as base. Fmoc protecting groups were removed using 50% (*v*/*v*) piperidine in DMF within 2 + 5 min. The products were cleaved from the solid support using a mixture of TFA:TIS (95:5) for 60 min, suspended in diethyl ether and purified by semipreparative HPLC. The products were usually isolated as solids after lyophilization.

For HPLC chromatography, a Dionex UltiMate 3000 system was used together with Chromeleon Software (version 6.80). For analytical and semipreparative chromatography, Chromolith Performance (RP-18e, 100–4.6 mm; Merck, Germany) and Chromolith semiprep (RP-18e, 100–10 mm; Merck, Germany) columns were used, respectively. For radioanalytical use, a Dionex UltiMate 3000 system equipped with a Raytest GABI Star radioactivity detector was used together with an analytical Chromolith Performance column (RP-18e, 100–4.6 mm; Merck, Germany). All operations were performed with a flow of 4 mL/min using H_2_O + 0.1% TFA and MeCN + 0.1% TFA as solvents.

Electrospray ionization (ESI) and matrix-assisted laser desorption/ionization (MALDI) and NMR spectra were obtained with Finnigan MAT95Q, Bruker Daltronics Microflex and Jeol AS500 spectrometers, respectively.

γ-Counting was performed using a 2480 Wizard gamma counter system from Perkin Elmer. A Cary 100 Bio system (Varian) was used to record the UV/Vis-Spectra together with 4 mL PMMA cuvettes from Sigma-Aldrich. Furthermore, for absorbance and emission measurements, a Tecan Infinite M200 Microplate reader together with a Nunc Micro-Well 96 solid plate from ThermoFisher was used.

Human embryonic kidney 293 cells stably expressing the GRP receptor (HEK-GRPR) were obtained from Dr. Martin Béhé, Paul Scherrer Institute; Villingen, Switzerland. Dulbecco’s modified Eagle’s medium (DMEM, high glucose, GlutaMax-I, 500 mL), geneticin (G418 Sulfate, 50 mg/mL), Opti-MEM I (GlutaMAX I), RPMI 1640 medium, L-glutamine and PenStrep were obtained from Gibco, fetal calf serum (FCS) from BioCell and Dulbecco’s phosphate buffered saline (PBS), 0.25% Trypsin and 0.02% EDTA solution in PBS from Sigma-Aldrich.

[^125^I]I-Tyr^4^-bombesin was purchased from Perkin Elmer (NEX258010UC, molar activity: 81.4 GBq/µmol). The ^68^Ge/^68^Ga-Generator used was an IGG100, obtained from Eckert & Ziegler, Berlin, Germany and eluted with HCl (0.1 M, 1.6 mL).

### 3.2. General Synthesis of the Target MIS **10**–**12** and **19**

Rink amide resin-Cys(Trt)-Lys(alloc)-NODA-GA(*t*Bu)_3_ was synthesized according to the standard Fmoc-based solid phase peptide synthesis protocols described earlier and the allyloxycarbonyl protecting group was removed still on solid support using tetrakis(triphenylphosphine)palladium(0) [31]. In the following step, 50 µmol Rink amide resin-Cys(Trt)-Lys-NODA-GA(*t*Bu)_3_ was reacted with the respective, HBTU-activated dye (100 µmol) at elevated temperature (80°C) in DMF (4 mL) for 3–4 h. The activation of the dyes was carried out with HBTU (0.95 eq.) and DIPEA (1.0 eq.) in DMF (2–4 mL) for 2 min. After the conjugation reaction was finished, the resin was filtered from the liquid components of the mixture and washed thrice with DMF, water, dichloromethane and diethylether. After drying, the dye conjugates were cleaved from solid support by using a mixture of TFA:TIS (95:5 (*v*/*v*), 5 mL) for 1–2 h. The volatile components were removed under reduced pressure and the residues were dissolved in 1:1 MeCN:H_2_O + 0.1% TFA and the products purified by semipreparative HPLC. Analytical data for each compound are given in the following, **10**: HPLC gradient: 0–100% MeCN + 0.1% TFA in 5 min, *R*_t_ = 2.80 min, yield: 30%, purity: 95%. MALDI-MS (*m*/*z*) for [M + H]^+^ (calculated): 1500.49 (1500.62), [M + Na]^+^ (calculated): 1522.48 (1522.61), [M + K]^+^ (calculated): 1538.49 (1538.59), [M + 2Na]^+^ (calculated): 1545.38 (1545.60). HR-ESI-MS (*m*/*z*) for [M + 2K + Na]^2+^ (calculated): 779.7598 (779.8108), **11**: HPLC gradient: 0–100 % MeCN + 0.1 % TFA in 5 min, *R*_t_ = 2.20 min, yield: 20%, purity: 95%. MALDI-MS (*m*/*z*) for [M + H]^+^ (calculated): 1480.46 (1480.56), [M + Na]^+^ (calculated): 1503.59 (1502.54), [M + K]^+^ (calculated): 1518.47 (1518.51). HR-ESI-MS (*m*/*z*) for [2M + 2K + 2Na]^2+^ (calculated): 1541.5837 (1541.5475), **12**: HPLC gradient: 0–100 % MeCN + 0.1 % TFA in 5 min, *R*_t_ = 2.00 min, yield: 5%, purity: 95%. MALDI-MS (*m*/*z*) for [M + H]^+^ (calculated): 1588.85 (1588.54), [M + Na]^+^ (calculated): 1610.84 (1610.53). HR-ESI-MS: detection of this compound by HR-ESI-MS was not successful. However, the respective peptide conjugate could be successfully identified, **19**: HPLC gradient: 0–100% MeCN + 0.1% TFA in 5 min, *R*_t_ = 2.10 min, yield: 70%, purity: 95%. MALDI-MS (*m*/*z*) for [M + H]^+^ (calculated): 534.91 (535.22), [M + Na]^+^ (calculated): 556.94 (557.20), [M + K]^+^ (calculated): 572.92 (573.17). HR-ESI-MS: detection of this compound by HR-ESI-MS was also not successful. Additionally, in this case, the respective peptide conjugate could however be successfully identified.

### 3.3. General Synthesis of Bioconjugates **14**–**16** and the Reference Compounds **17** and **18**

**13** (4 mg, 1.17 µmol) and **10**–**12**, **19** and **20** (1.3 µmol) were dissolved in 1:1 MeCN:H_2_O + 0.1% TFA (500 µL) and the pH of the mixture was adjusted to 7.0 with phosphate buffer (0.5 M, pH = 7.2). After 5 min, the crude products were purified by semipreparative HPLC, giving the products as green solids (**14**–**16**) or white powders (**17** and **18**), respectively. Analytical data for each compound are given in the following, **14**: HPLC gradient: 0–100% MeCN + 0.1% TFA in 12 min, *R*_t_ = 6.53 min, yield: 25%, purity: 99%. MALDI-MS (*m*/*z*) for [M + H]^+^ (calculated): 4904.96 (4904.20), [M + Na]^+^ (calculated): 4926.41 (4926.19), [M + K]^+^ (calculated): 4941.97 (4942.17), [M + 2H]^2+^ (calculated): 2453.13 (2452.92). HR-ESI-MS (*m*/*z*) for [M + 2Na + H]^3+^ (calculated): 1650.0109 (1650.0639), [M + 2Na + K]^3+^ (calculated): 1662.7185 (1662.7158), **15**: HPLC gradient: 0–100% MeCN + 0.1% TFA in 12 min, *R*_t_ = 4.20 min, yield: 30%, purity: 99%. MALDI-MS (*m*/*z*) for [M + H]^+^ (calculated): 4885.01 (4884.13), [M + Na]^+^ (calculated): 4905.84 (4906.12), [M + K]^+^ (calculated): 4925.11 (4922.09), [M + 2H]^2+^ (calculated): 2442.02 (2442.57). HR-ESI-MS (*m*/*z*) for [M + 3Na + K]^4+^ (calculated): 1248.2582 (1248.2863), [M + 2Na + 2K]^4+^ (calculated): 1251.5088 (1251.7592), **16**: HPLC gradient: 0–100 % MeCN + 0.1 % TFA in 12 min, *R*_t_ = 4.56 min, yield: 25%, purity: 99%. MALDI-MS (*m*/*z*) for [M + H]^+^ (calculated): 4993.96 (4992.12), [M + Na]^+^ (calculated): 5014.21 (5014.11), [M + K]^+^ (calculated): 5030.98 (5030.08), [M + 2H]^2+^ (calculated): 2497.96 (2496.56). HR-ESI-MS (*m*/*z*) for [M + 2H + Na + K]^4+^ (calculated): 1263.7520 (1263.7795), **17**: HPLC gradient: 0–100% MeCN + 0.1% TFA in 12 min, *R*_t_ = 4.21 min, yield: 80%, purity: 99%. MALDI-MS (*m*/*z*) for [M + H]^+^ (calculated): 3938.81 (3938.80), [M + Na]^+^ (calculated): 3960.58 (3960.78), [M + K]^+^ (calculated): 3976.34 (3976.76), [M + 2H]^2+^ (calculated): 1969.98 (1969.89). HR-ESI-MS (*m*/*z*) for [M + 3Na]^3+^ (calculated): 1335.2509 (1335.5978), **18**: HPLC gradient: 0–100% MeCN + 0.1% TFA in 12 min, *R*_t_ = 6.50 min, yield: 65%, purity: 99%. MALDI-MS (*m*/*z*) for [M + H]^+^ (calculated): 3451.23 (3452.58), [M + Na]^+^ (calculated): 3474.52 (3474.58), [M + H + K]^+^ (calculated): 3490.57 (3490.55), [M + 2H]^2+^ (calculated): 1726.29 (1726.29). HR-ESI-MS (m/z) for [M + 3H]^3+^ (calculated): 1151.5413 (1151.5365).

### 3.4. Radiochemistry

A solution of the respective MIS **10**–**12** or **19** or MIS bioconjugate **14**–**16** or **17** (5 nmol) in H_2_O (Tracepur^®^) was added to 90–120 MBq of [^68^Ga]GaCl_3_ obtained by fractioned elution of the ^68^Ge/^68^Ga generator system with HCl (0.1 M, 1.6 mL) and subsequent titration to pH 3.5–4.2 by addition of sodium acetate solution (1.25 M, 50–75 µL). All labeling experiments were performed in the presence of 2 mg ascorbic acid to suppress radiolysis-induced product fragmentation. When radiolabeling MIS **10**–**12** or **19**, TCEP × HCl (1–4 mg) was added to the reaction mixtures and the pH was again adjusted to 3.5–4.2 by addition of sodium acetate solution (1.25 M, 5–10 µL). After reaction for 10 min at 45 °C, the reaction mixtures were analyzed by analytical radio-HPLC. The radiolabeled products were found to be 95–99% pure and obtained in non-optimized molar activities of 90–120 GBq/µmol.

### 3.5. Determination of log_D_s

The water/1-octanol partition coefficients (log*_D_*) of [^68^Ga]Ga-**10**–[^68^Ga]Ga-**12**, [^68^Ga]Ga-**19**, [^68^Ga]Ga-**14** – [^68^Ga]Ga-**16** and [^68^Ga]Ga-**17** were determined by adding 5 µL of the respectively ^68^Ga-labeled compound (0.8–1.2 MBq, obtained as described before) to a mixture of phosphate buffer (0.05 M, pH = 7.4, 795 µL) and 1-octanol (800 µL). The mixtures were vigorously shaken for 5 min on a vibrating plate. After subsequent centrifugation at 13,000 rpm for 5 min, 125 µL was taken from each phase and measured in a γ-counter. The log*_D_* values were calculated from three or four independent experiments, each performed in triplicate. For **18**, the log*_D_* was determined by semipreparative HPLC. For this purpose, a solution of **18** (10 µL, c = 5 × 10^−4^ mol/L) in water was added to a mixture of 1 mL 1-octanol and 990 µL phosphate buffered solution (0.05 M, pH 7.4) and vigorously shaken for 5 min. After centrifugation, the phases were separated and both phases were analyzed by semipreparative HPLC and the peaks of the UV signals at 214 nm were integrated. The log*_D_* was determined by three separate measurements, each experiment performed in triplicate.

### 3.6. Competitive Receptor Binding Assay

Stably GRPR-transfected human embryonic kidney 293 cells (HEK-GRPR) were cultured at 37 °C in Dulbecco’s modified Eagle’s medium (DMEM, high glucose, GlutaMax-I, 500 mL) supplemented with 10% FCS (50 mL), 1.5% geniticin (8.25 mL) and 1% PenStrep (5.5 mL) in a humidified atmosphere containing 5% CO_2_. The medium was exchanged every two or three days and cells were split at >75% confluence. In vitro binding affinities were determined via competitive displacement experiments, which were performed at least trice, each experiment performed in triplicate. A Millipore Multiscreen punch kit and Millipore 96-well filter plates (pore size 1.2 µm) were used. The plates were incubated with PBS/BSA (1%) solution (each well 200 µL) for one hour before use. HEK-GRPR cells were harvested and suspended carefully in Opti-MEM I (GlutaMAX I) medium. Then, 50 µL of a cell suspension containing 10^5^ cells was seeded in each well. To this, a total volume of 50 µL was added to each well, containing the GRPR-specific radioligand [^125^I]I-Tyr^4^-bombesin (25 µL, 0.012 kBq/µL, 81.4 GBq/µmol) and the respective competitor **14**–**18** or endogenous bombesin (BBN, used as reference compound) (25 µL). The competitor was used in 11 different concentrations ranging from 0.25–500 nM for **14**–**18** or 0.1–250 nM for BBN, whereat the twelfth well contained no competitor to ensure 100% binding of the radioligand. After one hour of incubation at ambient temperature, the solution was filtrated, and the filters were washed with cold PBS (3 times). The filters were collected and measured by γ-counting. The 50% inhibitory concentration (IC_50_) values of **14**–**18** and BBN were calculated by fitting the obtained data via a nonlinear regression analysis using GraphPad Prism Software (v5.04).

## 4. Conclusions

We were able to develop a synthetic strategy enabling the design and synthesis of hybrid multimodal, chelator- and NIR dye-carrying imaging units that can be used for chemoselective and efficient introduction into biomolecules. The specific chelator and also the fluorescent dye are, however, not limited to the ones used here, allowing for a tailored design of the hybrid agents for biomolecule modification.

The developed MIS were shown to be efficiently introducible into a model homobivalent peptidic GRP receptor-specific carrier and the chemical, biological and photophysical characteristics of the conjugates were determined. These studies show that (i) the lipophilicity of the conjugates is mainly determined by the chosen cyanine dye; (ii) the radiolabeling of the hybrid multimodal imaging synthons and their bioconjugates is not influenced by the respective dye molecule; (iii) the photophysical properties of the parent dyes are not negatively affected upon introduction into MIS or their bioconjugates; and (iv) the target GRPR affinities were not considerably influenced by the introduction of a chelator or bulky and structurally complex chelator- and NIR dye-comprising MIS per se as could be shown for the multimodal bioconjugate comprising LS277 (**1**), showing only a minor alteration of binding parameters. In contrast, the use of the higher-charged cyanine analogs CK002 (**2**) and SK015 (**3**) resulted in conjugates with significantly decreased GRPR affinities.

Thus, the provided toolbox, comprising a synthetic strategy towards multimodal chelator- and NIR cyanine dye-modified building blocks furthermore carrying a thiol group for efficient and chemoselective biomolecule modification, enables the synthesis of tailored hybrid multimodal imaging synthons for dual biomolecule labeling with a radiometal nuclide and a NIR fluorescent dye. Through this, agents for combined multimodal imaging via PET and NIR-OI can be obtained, meeting the specific need and envisioned application of the imaging agent.

## Figures and Tables

**Table 1:** Summary of the chemical, biological and photophysical properties of the NIR fluorescent dyes **1–3**, the corresponding MIS **10**–**12** and **19** and the PESIN (PEG_3_-BBN_7–14_)-homodimer conjugates **14**–**18**. IC_50_ BBN: 2.81 ± 0.56 nM. Excitation wavelength λ_ex_ = 650 nm.

Compound	RCY ± SD (%)	log*_D_*	IC_50_ (nM)	λ_max(abs)_ (nm)	log ε (M^−1^ cm^−1^)	λ_max(em)_ (nm)	Stokes Shift (nm)
**1**	-	−1.03 ± 0.11	-	804	5.00	815	11
**10**	98.0 ± 2.1	−1.29 ± 0.11	-	720/790	4.80/4.61	800	10
**14**	98.3 ± 0.6	−1.33 ± 0.04	27.39 ± 2.01	735/810	4.57/4.61	820	10
**2**	-	−4.00 ± 0.11	-	761	5.44	781	20
**11**	98.3 ± 0.8	−3.05 ± 0.08	-	690/760	4.88/5.11	790	30
**15**	95.8 ± 0.2	−2.48 ± 0.14	56.07 ± 1.47	705/770	4.58/5.06	800	30
**3**	-	−3.95 ± 0.15	-	756	5.32	775	19
**12**	96.2 ± 0.6	−3.47 ± 0.17	-	690/760	4.83/5.30	790	30
**16**	96.8 ± 1.3	−2.55 ± 0.13	181.23 ± 2.45	700/770	4.50/5.07	790	20
**19**	99.0 ± 0.4	−3.35 ± 0.15	-	-	-	-	-
**17**	99.1 ± 0.8	−2.38 ± 0.03	21.48 ± 1.22	-	-	-	-
**18**	-	−0.99 ± 0.03	16.64 ± 1.06	-	-	-	-

## Supplementary Materials

The following are available online at https://www.mdpi.com/1424-8247/13/9/250/s1, Description of alternative synthesis pathways and associated experimental details. Scheme S1: Schematic depiction of the unsuccessful synthetic strategies towards the synthesis of the target multimodal imaging synthons (MIS), Scheme S2: Schematic depiction of the synthetic pathway producing different model MIS comprising derivatives of fluorescein, coumarin, dansyl and pyridinium dyes (8a–d)., Scheme S3: Schematic depiction of the alternative synthesis pathway towards 8b, utilizing the fluorescent dye-modified lysine derivative Fmoc-Lys(coumarin 343)-OH (9) during solid phase-assisted synthesis.
