# Supplementary material for: Hybrid Multimodal Imaging Synthons for Chemoselective and Efficient Biomolecule Modification with Chelator and Near-Infrared Fluorescent Cyanine Dye
Source: Pharmaceuticals (Basel). 2020 Sep 16;13(9):250. doi: 10.3390/ph13090250 (PMC7558102; doi:10.3390/ph13090250)
Supplement: Supplementary file 1 [file pharmaceuticals-13-00250-s001.pdf]

Supporting information for manuscript

Article

Hybrid Multimodal Imaging Synthons for Chemoselective and Efficient Biomolecule Modification with Chelator and Near-Infrared Fluorescent Cyanine Dye

Ralph Hübner [1,*], Valeska von Kiedrowski [2], Vanessa Benkert [3], Björn Wängler [2], Ralf Schirrmacher [4], Roland Krämer [3] and Carmen Wängler [1,*]

1 Biomedical Chemistry, Department of Clinical Radiology and Nuclear Medicine, Medical Faculty Mannheim of Heidelberg University, Theodor-Kutzer-Ufer 1-3, 68167 Mannheim, Germany
2 Molecular Imaging and Radiochemistry, Department of Clinical Radiology and Nuclear Medicine, Medical Faculty Mannheim of Heidelberg University, Theodor-Kutzer-Ufer 1-3, 68167 Mannheim, Germany; Valeska.vonKiedrowski@medma.uni-heidelberg.de (V.v.K.); Bjoern.Waengler@medma.uni-heidelberg.de (B.W.)
3 Institute of Inorganic Chemistry, Heidelberg University, Im Neuenheimer Feld 274, 69120 Heidelberg, Germany; Vanessa.mueller@aci.uni-heidelberg.de (V.B.); kraemer@aci.uni-heidelberg.de (R.K.)
4 Department of Oncology, Division of Oncological Imaging, University of Alberta, 11560 University Avenue, Edmonton, Alberta T6G 1Z2, Canada; schirrma@ualberta.ca
* Correspondence: Ralph.Huebner@medma.uni-heidelberg.de (R.H.); Carmen.Waengler@medma.uni-heidelberg.de (C.W.)

Content

Additional information regarding the initial, suboptimal synthetic attempts towards the target multimodal imaging synthons — page 2

Synthetic details for **5**, **6**, **7**, **8a–d** and **9** — page 4

References — page 5

Additional Information Regarding the Initial, Suboptimal Synthetic Attempts towards the Target Multimodal Imaging Synthons

In the first attempt, we used a Fmoc-Cys(Trt)-loaded Wang resin to conjugate first, Fmoc-Lys(Mtt)-OH and second (after N_ε-Mtt-deprotection under mild acidic conditions), the protected chelator NODA-GA(tBu)$_3$ in this N_ε-position, followed by conjugation of the respective 2-(1H-benzotriazol-1-yl)-1,1,3,3-tetramethyluronium hexafluorophosphate (HBTU)-activated near-infrared (NIR) dye (Scheme S1A). However, the target molecules could not be obtained after acidic cleavage from the resin and deprotection. Only compounds of low molecular weight not carrying the dye moieties were obtained.

As we assumed that this problem resulted from the dye conjugation on resin, we repeated the synthesis using standard reaction conditions (as detailed in the Materials and Methods section) but also varied solvent, coupling agent and reaction time (DMF→DMSO or NMP, HBTU→1-[Bis(dimethylamino)methylene]-1H-1,2,3-triazolo[4,5-b]pyridinium 3-oxid hexafluorophosphate (HATU) or Benzotriazole-1-yl-oxy-tris-pyrrolidino-phosphonium hexafluorophosphate (PyBOP), 30 min standard coupling time → up to 16h reaction time). We intended to cleave the intermediate **4** from the resin and to perform the conjugation of the NIR dyes in solution (Scheme S1B). Intermediate **4** could, however, not be obtained after cleavage from the resin and only products of lower molecular weight were isolated.

We hypothesized that this phenomenon was caused by the Lys-N_ε-Mtt-deprotection and chelator conjugation in this position on the resin. Hence, we first synthesized the NODA-GA-lysine-conjugate Fmoc-Lys(NODA-GA(tBu)$_3$)-OH (**5**) in solution and used it for the following solid-supported synthesis of **4** (Scheme S1C). Unfortunately, we were still not able to isolate **4** but again only products of lower molecular weight, which were not further characterized. This was unexpected as this synthetic approach comprised only one conjugation and one deprotection/cleavage step, and usually proceeds with high efficiency. It is thus unclear what were the reasons for the observed synthetic difficulties.

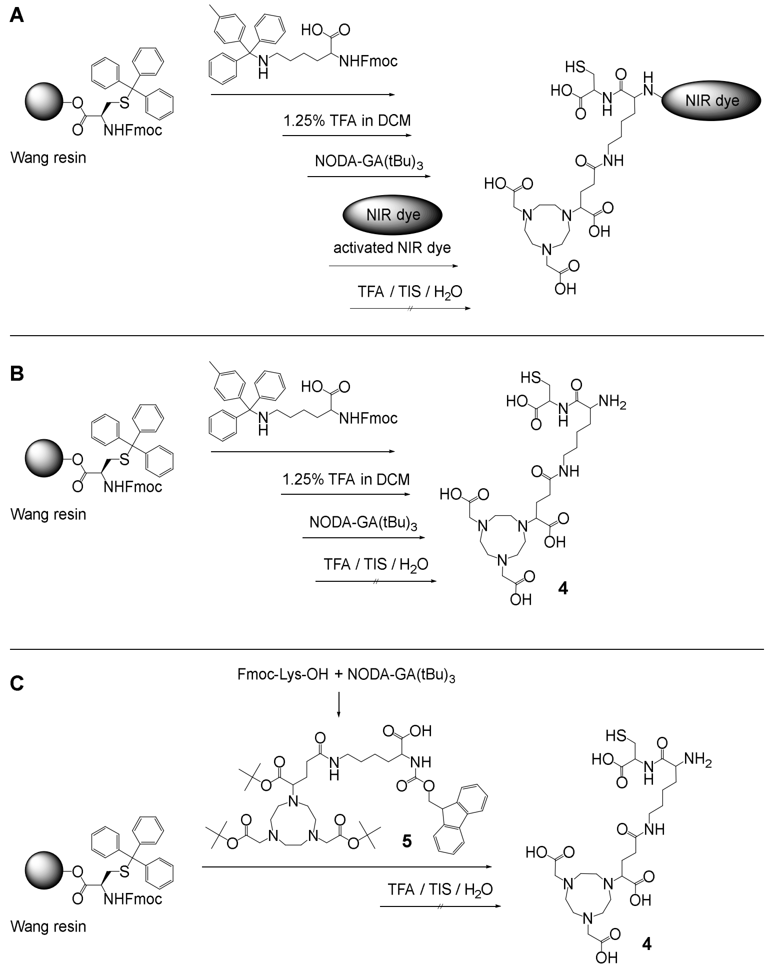

Scheme S1. Schematic depiction of the unsuccessful synthetic strategies towards the synthesis of the target multimodal imaging synthons (MIS). At first, standard reaction conditions were used (reaction in DMF for 30 min using 4 eq. of amino acid, 3.9 eq. of 2-(1H-benzotriazol-1-yl)-1,1,3,3-tetramethyluronium hexafluorophosphate (HBTU) as coupling reagent and 4 eq. of *N*,*N*-diisopropylethylamine (DIPEA) as base, Fmoc protecting groups were removed using 50% (v/v) piperidine in DMF within seven min and the products were cleaved from the solid support using a mixture of trifluoroacetic acid (TFA) : TIS triisopropylsilane (TIS) : H_2O (95:2.5:2.5) for 60 min) but also other solvents (DMSO, NMP), coupling agents (1-[Bis(dimethylamino)methylene]-1H-1,2,3-triazolo[4,5-b]pyridinium 3-oxid hexafluorophosphate: HATU, Benzotriazole-1-yl-oxy-tris-pyrrolidino-phosphonium hexafluorophosphate: PyBOP) and prolonged reaction times (up to 16h) were tested, giving the same results.

The reasons for these negative results are not obvious. We changed the cysteine-comprising solid support to a thio-ethanol amine-modified cysteamine 2-chlorotrityl resin, liberating the free thiol to see if this influences the synthesis outcome. Similar to before, no product formation could be observed. After further replacement of the resin with a highly acid-sensitive cysteamine super acid sensitive resin (SASRIN) resin and further change of the position of the chelator introduction from the Lys-N_ε side chain functionality to the N_α amino functionality, intermediate **6** could be obtained in at least moderate yields of 23%. Then, **6** could be further reacted with different activated model fluorescent dyes (5(6)-carboxyfluorescein-pfp ester [1], coumarin 343-pfp ester (**7**), dansyl chloride, and Py1 [2]), producing the respective model chelator-dye-conjugates **8a–d** in yields of 14–24% (Scheme S2).

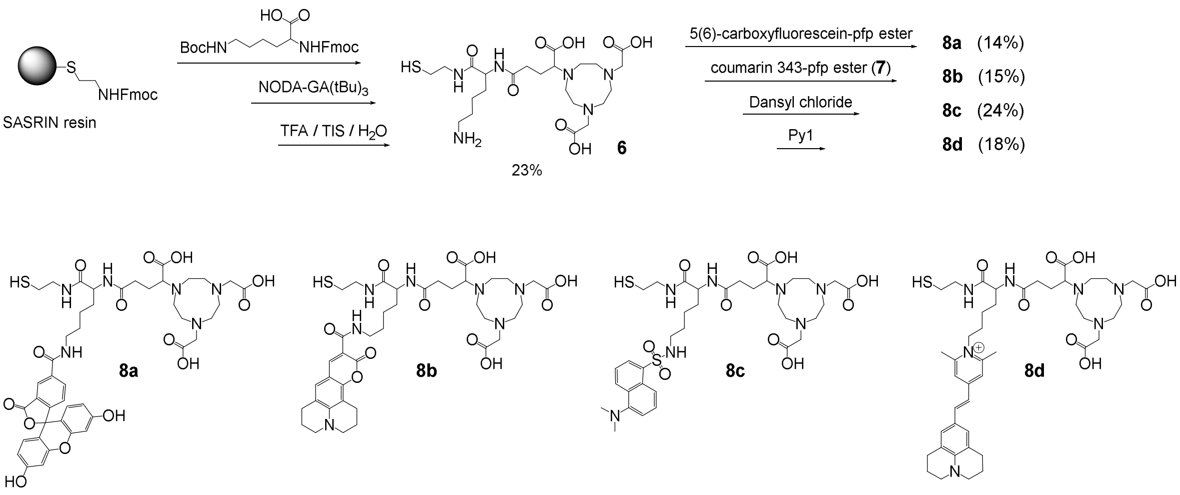

Scheme S2. Schematic depiction of the synthetic pathway producing different model MIS comprising derivatives of fluorescein, coumarin, dansyl and pyridinium dyes (**8a**–**d**).

The relatively low yields observed for the second synthesis step of **6** with the activated model dyes to **8a**–**d** following this route can be attributable to the presence of a second unprotected functional group (-NH_2 and -SH) being able to react with the activated dyes. Furthermore, the free thiol functionalities of the molecules **6** and **8a**–**d** can result in the formation of –S-S– bridges under neutral or slightly basic conditions, resulting in the formation of further side products.

To circumvent these problems and to determine if the complete synthesis of the multimodal imaging synthons (MIS) can be performed on resin, we further modified the synthesis route. Using coumarin 343 as a model, we studied the possibility to first conjugate the dye in its pfp ester form to the free Lys-N_ε amino functionality of Fmoc-Lys-OH to obtain Fmoc-Lys(coumarin 343)-OH (**9**) and to directly use this amino acid derivative in the synthesis of **8b** on solid support (Scheme S3). This procedure omits the cleavage of intermediate **6** from the resin and its purification prior to dye conjugation and should furthermore reduce the number of possible side products significantly. Indeed, this approach gave **8b** somewhat higher product yields of 24% instead of 15%. However, this relatively small increase in product yields does not seem to justify the additional synthetic efforts for the solution-phase preparation of the Fmoc-Lys(dye)-OH conjugates such as **9**.

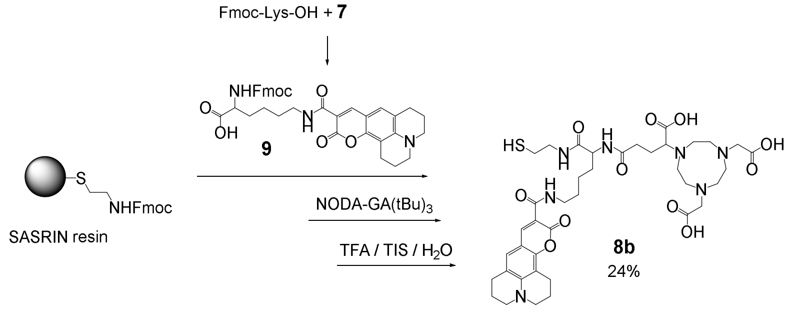

Scheme S3. Schematic depiction of the alternative synthesis pathway towards **8b**, utilizing the fluorescent dye-modified lysine derivative Fmoc-Lys(coumarin 343)-OH (**9**) during solid phase-assisted synthesis.

Thus, we tried another possibility to introduce the NIR dyes into the Lys-N_ε side chain amino functionality of the lysine building block, omitting a Mtt-deprotection of this functionality on resin by mild acids (as this might result in a loss of material to due a cleavage of the whole synthon from the solid phase) and not requiring the synthesis of the respective Fmoc-Lys(dye)-OH conjugates.

Details of this synthesis pathway, finally yielding the target MIS, are provided in the manuscript.

Chemical Syntheses of 5, 6, 7, 8a–d and 9

Fmoc-Lys(NODA-GA(*t*Bu)$_3$)-OH (5). To a solution of NODA-GA(*t*Bu)$_3$ (500 mg, 921 μmol) and HBTU (474 mg, 911 μmol) in DMF (5 mL) was added DIPEA (314 μL, 1.84 mmol) and the mixture was allowed to react for 2 min before Fmoc-Lys-OH (339 mg, 921 μmol) was added as solid. After 30 min, the mixture was acidified using neat TFA (200 μL) and the volatiles were evaporated. The residue was taken up in H_2O (25 mL) and the aqueous solution washed with diethylether (25 mL) thrice. The water was removed under reduced pressure. The product (645 mg, 722 μmol, 78%) was obtained as colorless hardening foam in a purity of 86% and used for subsequent reactions without further purification. A small portion was purified by semipreparative HPLC (HPLC gradient: 0–100 % MeCN + 0.1 % TFA in 5 min, R_t = 3.61 min) for analytic purposes. ^{1}H-NMR (500 MHz, methanol-*d*$_4$, 25°C) (*δ*, ppm; *J*, Hz): 7.88 (d, 2H, J^3=7.5), 7.73 (t, 2H, J^3=6.6), 7.42 (t, 2H, J^3=7.5), 7.35-7.26 (m, 3H), 6.78 (d, 1H, J^3=8.3), 4.38-4.18 (m, 4H), 3.95 (bs, 4H), 3.68 (t, 1H, J^3=7.3), 3.40 (q, 2H, J^3=7.0), 3.36-2.95 (m, 12H), 2.39 (t, 2H, J^3=7.1), 2.09-2.08 (m, 2H), 2.04-1.73 (m, 2H), 1.59-1.50 (m, 2H), 1.47 (s, 27H), 1.11 (t, 2H, J^3=7.0). ^{13}C-NMR (125 MHz, methanol-*d*$_4$, 25°C) (*δ*, ppm): 172.24, 157.03, 145.13, 142.08, 128.56, 127.98, 126.22, 120.84, 82.33, 67.14, 66.11, 56.21, 54.73, 51.62, 50.27, 47.99, 39.40, 32.87, 32.22, 28.31, 28.27, 26.56, 23.87. HR-ESI-MS (m/z) for $[M+H]^+$ (calculated): 894.5224 (894.5228), $[M+Na]^+$ (calculated): 916.5053 (916.5048).

Thioethanolamine-Lys-NODA-GA (6). The compound was assembled on solid support using standard Fmoc-based solid phase peptide synthesis protocols performed as described in the general part. The product was isolated as white solid after lyophilization. HPLC gradient: 0–100 % MeCN + 0.1 % TFA in 5 min, R_t = 1.35 min. HR-ESI-MS (m/z) for $[M+H]^+$ (calculated): 563.2857 (563.2863), $[M+Na]^+$ (calculated): 585.2684 (585.2683).

Coumarin 343-pfp ester (7). To a solution of coumarin 343 (490 mg, 1.72 mmol) and 2,3,4,5,6-pentafluorophenol (348 mg, 1.89 mmol) in chloroform (30 mL) was dropwise added a solution of dicyclohexylcarbodiimide (390 mg, 1.89 mmol) in pyridine (3 mL) and reacted for 3h at 60°C. The mixture was treated with water (25 mL) thrice and the organic layer was dried and the volatiles were evaporated. The product was purified by column chromatography using n-hexane : ethyl acetate 1:1 (R_f = 0.45) and obtained as dark orange solid in 25% yield (195 mg, 432 μmol). HPLC gradient: 0%–100 % MeCN + 0.1 % TFA in 5 min, R_t = 4.06 min. ^{1}H-NMR (C*D*Cl$_3$) (*δ*, ppm; *J*, Hz): 8.50 (s, 1H), 6.99 (s, 1H), 3.40 (q, 1H, J^3=5.6), 2.88 (t, 2H, J^3=6.3), 2.78 (t, 2H, J^3=6.1), 2.03-1.96 (m, 4H). ^{13}C-NMR (C*D*Cl$_3$) (*δ*, ppm): 160.04, 158.01, 154.12, 150.90, 149.99, 127.74, 119.93, 107.72, 105.74, 102.36, 50.50, 50.10, 27.35, 20.95, 19.95, 19.94. HR-ESI-MS (m/z) for $[M+H]^+$ (calculated): 452.0917 (452.0921).

Model MIS 8a–d. A solution of **6** (3.6 mg, 6.40 μmol) in H_2O (5 mL) was added to a solution of the respective dye derivative (5(6)-carboxyfluorescein-pfp ester, **7**, dansyl chloride or Py1, 1.1 eq each) in chloroform (5 mL), followed by DIPEA (5 μL, 29.3 μmol) and the mixtures were vigorously stirred overnight. The phases were separated, the aqueous phase was washed with chloroform (2 mL). The combined organic phases were dried and the solvent was evaporated. The crude product was dissolved in H_2O:MeCN 1:1 and purified by semipreparative HPLC, giving the products in yields of 14–24%. Analytical data for each compound are given in the following, **8a** (5(6)-carboxyfluorescein): HPLC gradient: 0–100% MeCN + 0.1 % TFA in 5 min, R_t = 2.50 min. HR-ESI-MS (m/z) for $[M+H]^+$ (calculated): 921.3351 (921.3340), **8b** (coumarin 343): HPLC gradient: 0%–100% MeCN + 0.1 % TFA in 5 min, R_t = 3.00 min. HR-ESI-MS (m/z) for $[M+H]^+$ (calculated): 830.3765 (830.3759), **8c** (dansyl): HPLC gradient: 0–100% MeCN + 0.1 % TFA in 5 min, R_t = 2.53 min. HR-ESI-MS (m/z) for $[M+H]^+$ (calculated): 796.3386 (796.3374), $[M+Na+K+formic\ acid]^+$ (calculated): 903.3654 (903.2890), **8d** (Py1): HPLC gradient: 0–100% MeCN + 0.1 % TFA in 5 min, R_t = 3.03 min. HR-ESI-MS (m/z) for $[M-H]^-$ (calculated): 849.4446 (849.4453), (m/z) for $[M-3H+2Na+K]^-$ (calculated): 932.3930 (932.3729).

Fmoc-Lys(coumarin 343)-OH (9). To a solution of **7** (150 mg, 333 μmol) and DIPEA (57 μL, 333 μmol) in chloroform (20 mL) was added first Fmoc-Lys-OH (122 mg, 333 μmol) and afterwards H_2O (5 mL). The mixture was stirred vigorously for 1h. The phases were separated and the aqueous solution was extracted with chloroform (10 mL) twice. The combined organic layers were washed with H_2O (5 mL), dried and the solvent was evaporated. The product was obtained in pure form in 63% yield (134

mg, 211 μmol). HPLC gradient: 0–100% MeCN + 0.1 % TFA in 5 min, R_t = 3.89 min. ^{1}H-NMR (C*D*Cl$_3$) (*δ*, ppm; *J*, Hz): 8.84 (bt, 1H, J^3=5.9), 8.55 (s, 1H), 7.73 (d, 2H, J^3=7.4), 7.64 (d, 2H, J^3=7.4), 7.37 (t, 2H, J^3=7.4), 7.29 (bt, 2H, J^3=7.6), 6.98 (s, 1H), 6.93 (bs, 1H), 5.09 (bd, 1H, J^3=7.6), 4.34 (t, 1H, J^3=7.1), 4.26-4.23 (m, 2H), 3.30-3.26 (m, 4H), 3.00 (q, 2H, J^3=7.4), 2.84 (t, 2H, J^3=6.4), 2.73 (t, 2H, J^3=6.1), 1.98-1.89 (m, 4H), 1.86-1.83 (m, 2H), 1.29-1.04 (m, 4H). ^{13}C-NMR (C*D*Cl$_3$) (*δ*, ppm): 165.88, 160.46, 153.63, 151.89, 147.11, 144.27, 142.34, 135.80, 128.20, 127.46, 126.96, 125.97, 125.49, 119.16, 119.77, 116.42, 107.06, 106.01, 67.94, 52.85, 50.03, 49.59, 41.46, 30.69, 30.29, 27.40, 26.14, 25.32, 24.63. HR-ESI-MS (m/z) for [M-H]$^-$ (calculated): 634.2563 (634.2553).

References

1. Adamczyk, M.; Fishpaugh, J.R.; Heuser, K.J. Preparation of succinimidyl and pentafluorophenyl active esters of 5- and 6-carboxyfluorescein. *Bioconjug. Chem.* **1997**, *8*, 253-255, doi:10.1021/bc9600877.
2. Wetzl, B.K.; Yarmoluk, S.M.; Craig, D.B.; Wolfbeis, O.S. Chameleon labels for staining and quantifying proteins. *Angew. Chem. Int. Ed.* **2004**, *43*, 5400-5402, doi:10.1002/anie.200460508.
